# In Silico Drug Design of Benzothiadiazine Derivatives Interacting with Phospholipid Cell Membranes

**DOI:** 10.3390/membranes12030331

**Published:** 2022-03-17

**Authors:** Zheyao Hu, Jordi Marti

**Affiliations:** Department of Physics, Polytechnic University of Catalonia-Barcelona Tech, B4-B5 Northern Campus UPC, 08034 Barcelona, Spain; zheyao.hu@upc.edu

**Keywords:** benzothiadiazine derivatives, drug design, molecular dynamics, phospholipid membrane

## Abstract

The use of drugs derived from benzothiadiazine, a bicyclic heterocyclic benzene derivative, has become a widespread treatment for diseases such as hypertension, low blood sugar or the human immunodeficiency virus, among others. In this work we have investigated the interactions of benzothiadiazine and four of its derivatives designed in silico with model zwitterionic cell membranes formed by dioleoylphosphatidylcholine, 1,2-dioleoyl-*sn*-glycero-3-phosphoserine and cholesterol at the liquid–crystal phase inside aqueous potassium chloride solution. We have elucidated the local structure of benzothiadiazine by means of microsecond molecular dynamics simulations of systems including a benzothiadiazine molecule or one of its derivatives. Such derivatives were obtained by the substitution of a single hydrogen site of benzothiadiazine by two different classes of chemical groups, one of them electron-donating groups (methyl and ethyl) and another one by electron-accepting groups (fluorine and trifluoromethyl). Our data have revealed that benzothiadiazine derivatives have a strong affinity to stay at the cell membrane interface although their solvation characteristics can vary significantly—they can be fully solvated by water in short periods of time or continuously attached to specific lipid sites during intervals of 10–70 ns. Furthermore, benzothiadiazines are able to bind lipids and cholesterol chains by means of single and double hydrogen-bonds of characteristic lengths between 1.6 and 2.1 Å.

## 1. Introduction

Plasma membranes are fundamental in the behavior of human cells, being not only responsible for the interactions between the cell and its environment but also for processes such as cellular signaling [1], enzyme catalysis [2], endocytosis [3] and transport, among others. The main structure of the cell membrane is composed of bilayer phospholipids including sterols, proteins, glycolipids and a wide variety of other biological molecules. High compositional complexity and versatility of membranes are closely related to the environment and the physiological state of cells [4,5] so that many diseases such as cancer, cardiopathies, diabetes, atherosclerosis, infectious diseases or neurodegenerative pathologies are accompanied by changes in the composition of cell membranes [6,7,8,9]. For such a reason, the knowledge of the behavior of drugs interacting with different membrane components and their distribution in damaged tissues maybe key to improving drug efficiency and the therapy of the diseases and it has become a topic of greatest scientific interest.

It is well known that the composition of cell membranes in different tissues and organs of the human body exhibits large variations. In the treatment of diseases, an efficient drug design could enhance the interaction of active pharmaceutical ingredients with membrane components in specific tissues helping to reach the target site successfully. Thus, there is a great demand for a full understanding of the rules of drug–membrane interactions, which may help us predict the distribution and curative effect of drugs in the body when it comes to the designing and testing of new drug molecules. Generally, medicinal chemists tended to overcome the difficulty of drugs in entering cells or crossing biological barriers, such as the blood–brain barrier [10,11,12] by modifying their structures to enhance the lipophilicity of drugs. However, little research has been performed on the influence of drug structure on the rule of drug–membrane interaction, notably the direct information on atomic interactions of drug-membrane systems at the all-atom level. In this work we establish a procedure for the in silico design of derivatives of the well-known family of benzothiadiazines.

Heterocyclic compounds are ubiquitous in the structure of drug molecules [13,14] playing an important role in human life [15,16]. Such compounds are common parts of commercial drugs having multiple applications based on the control of lipophilicity, polarity and molecular hydrogen bonding capacity. Among them, benzothiadiazine and its derivatives have wide pharmacological applications, such as diuretic [17], anti-viral [18], anti-inflammatory [19], regulating the central nervous system [20] and, more recently, as anti-cancer agents [21,22,23]. In addition to the above-mentioned biopharmacological activities, benzothiadiazine derivatives also have bio-activity such as Factor Xa inhibition [24], anti-Mycobacterium [25,26] and anti-benign prostatic hyperplasia [27]. 3,4-dihydro-1,2,4-benzothiadiazine-1,1-dioxide (DBD) being the main common structure of the benzothiadiazine family was investigated in a previous work [28] to elucidate the mechanisms responsible for the interactions of DBD with the basic components of cell membranes in all-atom level for the first time. In the present work, our aim was to design in silico DBD derivatives that may be employed with the purpose of inhibiting a limited variety of tumors produced by the oncogenic protein KRas-4B (such as pancreatic, lung or colorectal [29,30]), work currently in progress in our lab. For such a purpose, it has been found convenient to model the substrate cell membrane with DOPC/DOPS lipids, since these particular components are most relevant for the absorption of the oncogene at the cell membrane’s interface (see for instance [31,32]). Further, in an effort to produce a more realistic setup, we decided to include cholesterol in the membrane model. Cholesterol constitutes about 33.3% of the outer leaflet in healthy colorectal cells [33], which is in good agreement with the 30% of cholesterol adopted in this work. We have already observed [28] that DBD has a strong affinity to the DOPC species of lipids and that it is also able to bind other membrane components by single and double hydrogen bonds. In this paper, we modified DBD and evaluated the effect of different substitutes on the affinity of the DBD to cell membrane components.

## 2. Methods

**Figure 1:**
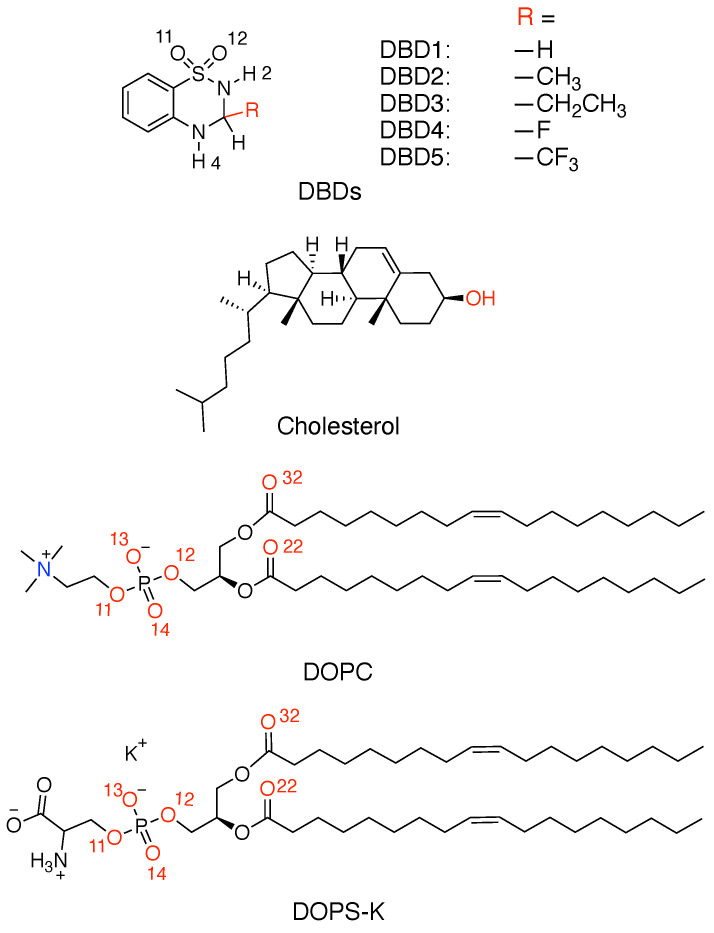
Chemical structures of benzothiadiazine derivatives, phospholipids and cholesterol. Site ‘R’ stands for the five DBD derivatives considered in the present work.

Five models of lipid bilayer membranes in aqueous solution have been constructed using the CHARMM-GUI web-based tool [34,35]. The membrane components and the amount of particles of each class are as follows: all systems include one single DBD derivative, 112 neutral DOPC lipids, 28 DOPS associated with K^+^ (DOPS-K) lipids, 60 cholesterol molecules, 49 potassium ions, 21 chlorine ions and 10,000 water molecules. The lipids have been distributed in two symmetric leaflets embedded inside an electrolyte potassium–chloride solution at 0.15 M concentration. We have considered five different setups, where only the benzothiadiazine derivative is different in each case. We considered a previously investigated [28] standard DBD species as the reference (DBD1) and four more DBD derivatives (DBD2, DBD3, DBD4 and DBD5), designed by ourselves using in silico techniques. The way we designed the new DBD species followed the fact that medicinal chemists modify the chemical structure of the drug for the purpose of improving its therapeutic effect, reducing toxicity and side effects. The modification method depends on the structure of the drug. Generally, when performing the structure modification, the basic structure of the drug will remain unchanged and only some functional group will change. When the drug acts, the binding methods of drug and receptor form a reversible complex and are generally by ionic bond, hydrogen bond or covalent bond. In a previous work [28] we observed that DBD can form hydrogen bonds (HB) and become absorbed by the cell membrane with DBD having strong affinity for DOPC. ‘H2’ and ‘H4’ sites of DBD are important for the formation of such HB with membrane components. The ‘R’ site (shown in Figure 1) is very close to the ‘H2’ and ‘H4’ sites so that the size, electronegativity and other properties of the R substituent will affect the ability of ‘H2’/‘H4’ to form hydrogen bonds with cell membrane components. So, with the tool of CHARMM-GUI platform “Ligand Reader & Modeller”, we introduced methyl, ethyl, fluorine and trifluoromethyl into this site in order to assess the effect of new drug structures on the behavior of DBD in cell membranes.

Sketches of all species are reported in Figure 1. Each DBD species and each phospholipid was described with atomic resolution (DBD1 and DBD4 have 20 sites, DBD2 and DBD5 have 23 sites, DBD3 has 26 sites, DOPC has 138 sites, DOPS has 131 sites and cholesterol has 74 sites). In all simulations water has been represented by rigid 3-site TIP3P [36] molecules, included in the CHARMM36 force field [37,38], that was adopted for lipid–-lipid and lipid–-protein interactions. In particular, we selected the version CHARMM36m [39], which is able to reproduce the area per lipid for the most relevant phospholipid membranes in excellent agreement with experimental data. The parameterization of the DBD species was performed by means of the “Ligand Reader & Modeller” tool in CHARMM-GUI platform (https://charmm-gui.org/?doc=input/ligandrm, accessed on 31 December 2021). All bonds involving hydrogen atoms were set to fixed lengths, allowing fluctuations of bond distances and angles for the remaining atoms. Van der Waals interactions were cut off at 12 Å with a smooth switching function starting at 10 Å. Finally, long-ranged electrostatic forces were computed using the particle mesh Ewald method [40], with a grid space of 1 Å, updating electrostatic interactions every time step of the simulation runs.

Molecular dynamics (MD) simulations have been revealed to be a very reliable tool for the simulation of the microscopic structure and dynamics of all sorts of condensed systems, such as aqueous solutions in bulk or under confinement [41,42,43,44,45,46,47] towards model cell membranes in electrolyte solution [48,49,50] and, more recently, small-molecule and protein systems attached to phospholipid membranes [51,52,53]. Five sets of MD runs were performed by means of the GROMACS2021 simulation package [54,55,56,57,58]. We run all the simulations at the fixed pressure of 1 atm and at the temperature of 310.15 K, typical of the human body and also well above the crossover temperatures for pure DOPC and DOPS needed to be at the liquid crystal phase (253 and 262 K, respectively) [59]. In all cases, the temperature was controlled by a Nose–Hoover thermostat [60] with a damping coefficient of 1 ps^−1^, whereas the pressure was controlled by a Parrinello–Rahman barostat [61] with a damping time of 5 ps. In the isobaric–-isothermal ensemble, i.e., under the condition of a constant number of particles, pressure and temperature, equilibration periods for all simulations were around 200 ns. In all cases, we recorded statistically meaningful trajectories of 600 ns. The simulation boxes had the same size in all cases, i.e., 78.1 × 78.1 × 95.7 Å^3^. We have considered periodic boundary conditions in the three directions of space. The simulation time step was fixed to 2 fs in all cases.

## 3. Results and Discussion

### 3.1. Characteristics of the Bilayer Systems

**Figure 2:**
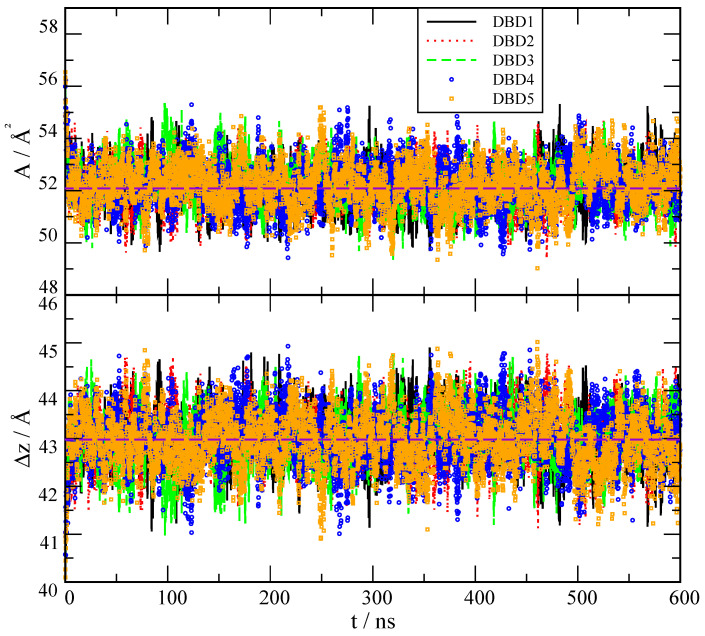
Area per lipid *A* and thickness Δz of the membrane systems including DBD derivatives as a function of simulation time *t*. DBD1 (continuous line); DBD2 (dotted line); DBD3 (dashed line); DBD4 (circles); DBD5 (squares). Long-dashed (purple) lines indicate the average values reported in Table 1.

The phospholipid bilayer considered in this work was previously simulated and its main characteristics were reported [28,30]. We found reliable values of the area per lipid *A* and the thickness Δz of the membranes to be in qualitative agreement with available experimental data. In order to corroborate these results in the present work where the system contains DBD derivatives, we computed *A* and Δz as usual, considering the total surface along the XY plane (plane along the bilayer surface) divided by the number of lipids and cholesterol in one single leaflet [62] and the difference between the *z*-coordinates of the phosphorus atoms of the two leaflets, respectively. The results of the averaged values obtained from the 600 ns production runs are reported in Table 1, whereas the time evolution of both properties is displayed in Figure 2.

The results shown in Figure 2 indicate that the simulated trajectories were well equilibrated in all cases. The comparison with previous results indicates that the effect of DBD derivatives on the area per lipid and thickness of the membrane is totally marginal. Firstly, the averaged result of A=52.2 Å${}^{2}$ in all cases matches perfectly the previous reported value of 52.0 Å${}^{2}$ [30] (where a large protein was embedded in the system) and also the experimental value of 54.4 Å${}^{2}$ reported by Nagle et al. [63]. Area/lipid shows fluctuations around 5% of the averaged values. Secondly, thickness of the membranes are also in good qualitative agreement with previous works: from Figure 2 we observe fluctuations less than 5% of the averaged values, of around 43.0 Å, as expected. Such value is in qualitative agreement with the experimental measurement of Δz = 40 Å for the DOPC-cholesterol (30%) bilayer, as reported by Nagle et al. [63] and it matches the previously found Δz = 43.0 Å obtained in previous simulations for the DOPC/DOPS/cholesterol membrane [30].

### 3.2. Local Structure of Benzothiadiazine Derivatives

#### 3.2.1. Radial Distribution Functions

We considered the so-called atomic pair radial distribution functions (RDF) $g_{AB}\left(r\right)$, defined, in a multicomponent system, for a species *B* close to a tagged species *A* as:

(1) $$g_{AB}\left(r\right)=\frac{V\;\langle n_{B}\left(r\right)\rangle }{4\;N_{B}\;\pi r^{2}\;\Delta r},$$

where $n_{B}\left(r\right)$ is the number of atoms of species *B* surrounding a given atom of species *A* inside a spherical shell of width Δr. *V* is the total volume of the system and $N_{B}$ is the total number of particles of species *B*. The physical meaning of the RDF stands for the probability of finding a particle *B* at a given distance *r* of a particle *A*. Our RDF are normalized so that tend to 1 at long distances, i.e., when the local density equals the averaged one.

**Figure 3:**
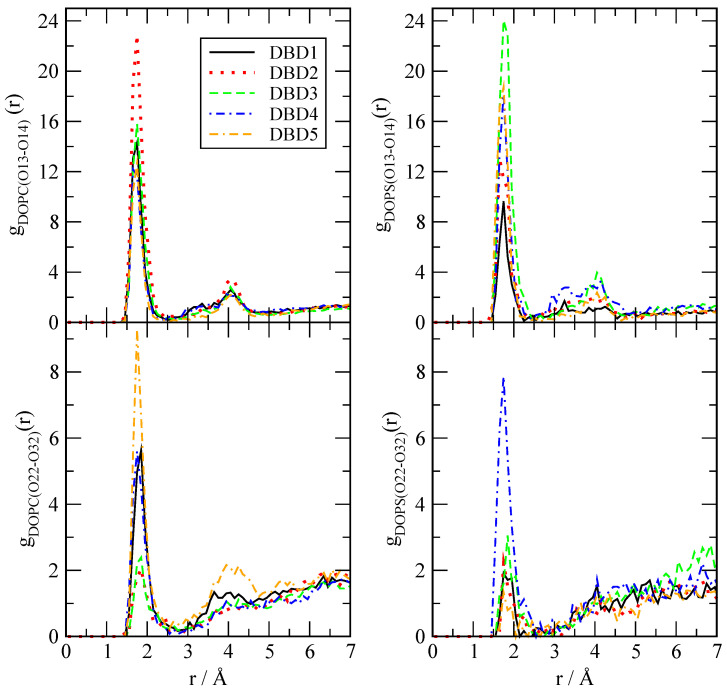
Radial distribution functions between site ‘H2’ of DBD derivatives and selected oxygen sites in DOPC and DOPS phospholipids. Sites ‘O13-14’ stand for head groups of the cell membrane phospholipids and sites ‘O22-32’ stand for tail groups located deeper in the membrane interface.

**Figure 4:**
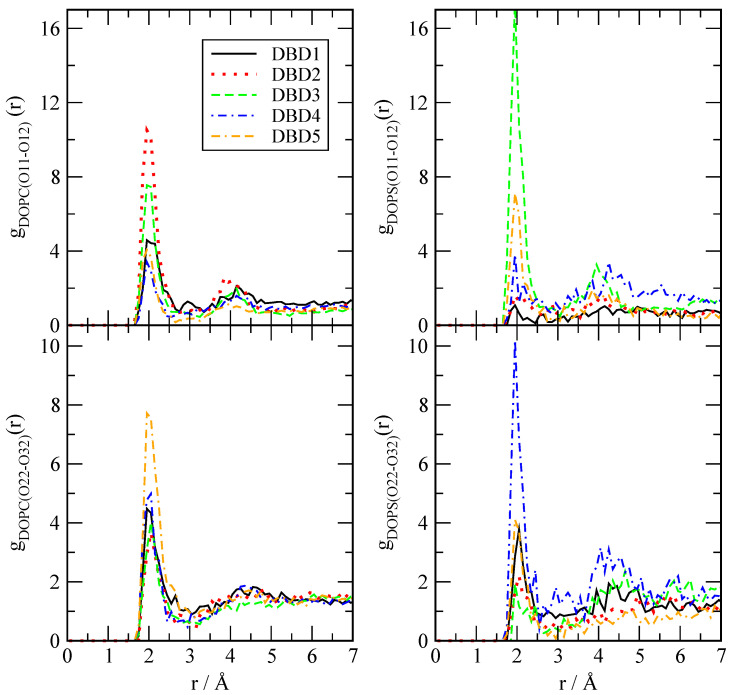
Radial distribution functions between site ‘H4’ of DBD derivatives and selected oxygen sites in DOPC and DOPS phospholipids.

**Figure 5:**
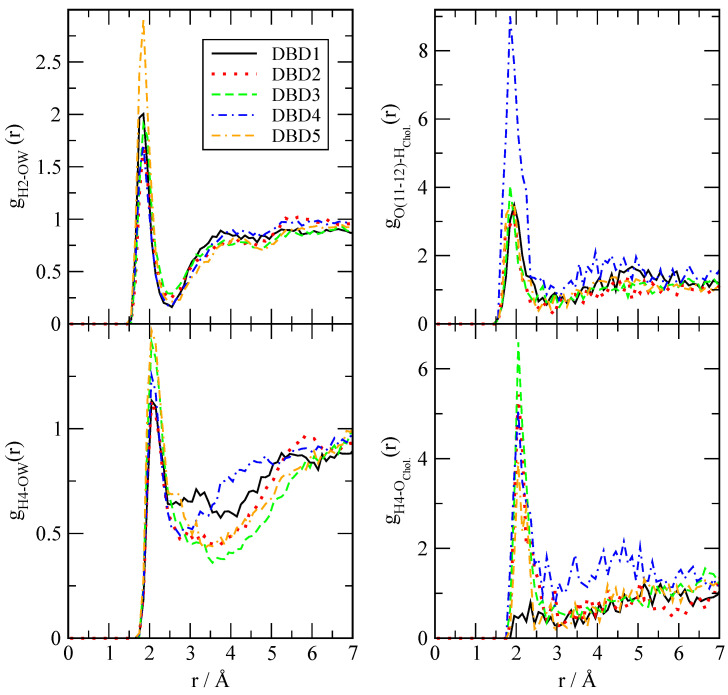
Radial distribution functions between sites ‘H2’ and ‘H4’ of DBD derivatives and selected sites of water (left column) and cholesterol (right column).

We have evaluated the local structure of the DBD derivatives when solvated by lipids, cholesterol and water according to Equation (1). Only a few of all possible RDF are reported, since we have selected the most relevant ones for the purpose of highlighting the main interactions between the tagged particles. The results are presented in Figure 3, Figure 4 and Figure 5, where we have selected the hydrogen sites ‘H2’, ‘H4’, ‘O11’ and ‘O12’ of DBD derivatives, since these are the most active sites, able to form hydrogen bonds with the surrounding partners (lipid, cholesterol species and eventually water). In all cases we can observe a clear first coordination shell associated to the binding of DBD derivatives to the membranes, with corresponding maxima indicating the typical HB distances, together with much lower second shells centered around 4–5 Å. As a general fact, the HB detected cover a noticeably wide range of distances, between 1.6 and 2.1 Å.

The structure of DBD derivatives described by their ‘H2’ site (see Figure 1) indicates the existence of HB formed by ‘H2’ and several sorts of lipid oxygen sites and it is represented in Figure 3. We can notice that the typical HB length is of 1.7 Å in all cases, both for the binding with oxygen atoms of the phosphoryl group ‘O13-14’ (located at the head groups of DOPC and DOPS, with both oxygen sites sharing a negative charge) and for the binding with sites ‘O22-32’ (located in the tail groups of the lipids) as well. This is the typical distance of the binding of small-molecules to cell membranes, such as tryptophan to dipalmytoilphosphatidylcholine (see for instance the review [64]). It should be pointed out that using fluorescence spectroscopy, Liu et al. [65] obtained values for the HB lengths of tryptophan-water between 1.6 and 2.1 Å, i.e., of the same range as those reported here.

This indicates that: (1) all sorts of DBD derivatives can bind the membranes at both head and tail groups and (2) depending on the oxygen sites, some derivatives are able to create HB stronger than others; however, the strength of the HB binding is not uniform and it clearly depends of the class of derivative and lipid chain involved. Despite we will qualitatively analyze the strength of the HB in Section 3.2.2, we can give some general clues here. For instance, DBD2 is able to bind DOPC more strongly than DBD1 (species that we will consider as the reference), with the remaining derivatives making bonds of similar strength. Nevertheless, when DOPS is concerned, all derivatives form stronger HB than DBD1, with DBD3 the strongest. Similar trends are observed when the internal tail group sites ‘O22-32’ are analyzed: DBD5 makes the strongest HB with DOPC and DBD4 makes the strongest bond with DOPS. In this latter case, the enhancement of the HB is milder than it occurred in the former case (head group bindings). Overall, we can observe that the one-site modifications proposed with the design of the new DBD-derivatives reported in this work has produced significant changes and enhancement of the HB connections to the model cell membrane. Generally speaking, electron-donating groups (DBD2-DBD3) produce similar qualitative effects on the DBD–membrane hydrogen bond connections, whereas electron-accepting types (DBD4-DBD5) tend to produce opposite effects. This can be valuable information to assess the affinity of new designed drugs to target specific oncogenes such as KRas-4B, work that it is been currently developed in our group.

Concerning hydrogens ‘H4’ of DBD derivatives and their binding characteristics when associated to DOPC and DOPS (Figure 4), we observed that they can be also connected either to ‘O11’ or ‘O12’ of the phospholipids (head groups), either to ‘O22’ or ‘O32’ (tail groups). Interestingly, in the case of DBD’s ‘H4’, HB lengths are within the range of 2–2.1 Å, significantly longer than those formed by H2 (range around 1.6 to 1.8 Å). This was already observed for the reference DBD1 in a previous work [28]. In this case, the strongest HB is observed when ‘H4’ of DBD3 is connected to DOPS’s ‘O11-12’ oxygens. In this particular case, the new DBD derivatives have shown to be able to bind the internal regions of the membrane, whereas the original benzothiadiazine species (DBD1) had a very low probability to penetrate these regions. Again for the ‘H4’ binding site, we have found a general enhancement of the binding of DBD derivatives with the main phospholipids forming our cell membrane system.

In the third RDF set (Figure 5) we report interactions between sites ‘H2’, ‘H4’ and ‘O11-12’ of DBD with water (plots at the left column) and cholesterol (plots at the right column). In the case of water, HB can be established between ‘H2’ and the oxygen site of water (top) or, alternatively, between ‘H4’ and the oxygen of water (bottom). In both cases, the strength of the interaction is low, which suggests that DBD derivatives are strongly bound to the cell membrane and can be solvated by a few water molecules located at the interface. We have not observed long term episodes of DBD derivatives fully solvated by water.

We have located some extent of hydrogen-bonding between DBD and cholesterol. However, no significant binding of ‘H2’ with cholesterol has been observed, whereas interactions of both ‘H4’ and ‘O11-12’ sites of DBD derivatives have been detected. In particular, the strongest contributions are seen for with hydroxyl’s oxygens of cholesterol with ‘H4’ of the DBD species, which were undetected for the reference original DBD1 as well as for oxygens of the DBD derivatives with hydroxyl’s hydrogen of cholesterol. In the latter case, we found a particularly strong contribution of DBD4, i.e., the derivative containing a fluoride residue instead the original hydrogen atom. The HB lengths are in the range of 2.1 Å in all cases.

#### 3.2.2. Potentials of Mean Force between Benzothiadiazine Derivatives and Lipids

Among the wide variety of one-dimensional free-energy methods proposed to compute the potential of mean force (PMF) between two tagged particles [66] a simple but meaningful choice is to consider the radial distance *r* as an order parameter, able to play the role of the reaction coordinate of the process, within the framework of unbiased simulations as those reported in the present work and to proceed with a direct estimation of the reversible work as described below. This has become one of standard choices to compute free-energy barriers in MD simulations, together with constrained MD simulations [67] or the popular *umbrella sampling* procedure [68]. In case that more accurate values of the free-energy barriers are needed, the optimal choices are: (1) to use constraint-bias simulation combined with force averaging for Cartesian or internal degrees of freedom [66]; (2) the use of multi-dimensional reaction coordinates [69] such as transition path sampling [70,71,72] or (3) considering collective variables, such as metadynamics [30,73] although such methods require a huge amount of computational time. Since the determination of reaction coordinates for the binding of DBD at zwitterionic membranes is out of the scope of this work, we limit ourselves to use radial distances between two species as our order parameters to perform reversible work calculations.

**Figure 6:**
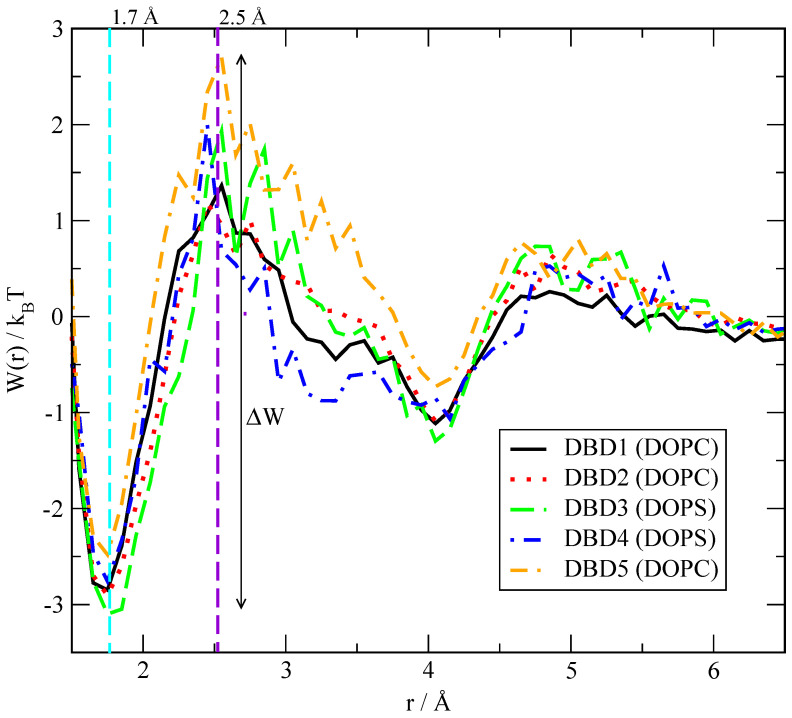
Potentials of mean force for the binding of ‘H2’ sites of DBD derivatives to the sites ‘O13-14’ of DOPC and DOPS.

In this framework, a good approximation of the PMF can be obtained by means of the reversible work $W_{AB}\left(r\right)$ required to move two tagged particles (A,B) from infinite separation to a relative separation *r* (see for instance Ref. [74], chapter 7):

(2) $$W_{AB}\left(r\right)=-\frac{1}{\beta }\ln g_{AB}\left(r\right),$$

where $\beta =1/(k_{B}T)$ is the Boltzmann factor, $k_{B}$ the Boltzmann constant and *T* the temperature. In the calculations reported here, the radial distance *r* is the distance used in the corresponding RDF (Section 3.2) i.e., it is not related to the atom position relative to the center of the membrane. All free-energy barriers are simply defined (in $k_{B}T$ units) by a neat first minimum and a first maximum of each W(r), with barrier size ΔW obtained as the difference between the former. As a sort of example, we present the free-energy barriers with largest values for each DBD species in Figure 6.

The full set of free-energy barriers for a wide selection of bound pairs has been reported in Table 2. There we can observe overall barriers between 1.2 and 5.2 $k_{B}T$, which correspond to 0.7–3.1 kcal/mol, for the simulated temperature of 310.15 K. We observe stable binding distances (given by the position of the first minima of the PMF) matching the typical hydrogen-bond distances, as expected. As a reference, it is known that the typical energy of water–water hydrogen-bonds estimated from ab initio calculations is of 4.9 kcal/mol for a water dimer in vacuum [75], whereas in our model system (including TIP3P water) the barrier associated to the HB signature, given by the first maximum of water’s oxygen–hydrogen RDF, is of 1.1 kcal/mol. This low value can be directly associated with two facts: (1) first, we have estimated this energy in the bulk, condensed phase of the aqueous ionic solvent, whereas the reference value of Feyereisen et al. [75] corresponds to an isolated water dimer, i.e., can be related to gas phase; (2) secondly, the TIP3P water model included in the CHARMM36 force field is well known to have significant drawbacks to describe liquid water [76].

In an earlier work [28] we reported by the first time DBD–membrane related free-energy barriers. For the sake of comparison with other similar systems, we can remark that the PMF of tryptophan in a di-oleoyl-phosphatidyl-choline bilayer membrane shows a barrier of the order of 4 kcal/mol [77], whereas the barrier for the movement of tryptophan attached to a poly-leucine α-helix inside a DPPC membrane was reported to be of 3 kcal/mol [78]. Finally, neurotransmitters such as glycine, acetylcholine or glutamate were reported to show small barriers of about 0.5–1.2 kcal/mol when located close to the lipid glycerol backbone [79]. These values could further indicate that our estimations match well the order of magnitude of the free-energy barriers for other small-molecules of similar size.

We designed two sets of DBD derivatives according to their characteristics: in DBD2 and DBD3 we replaced a hydrogen by electron-donating groups (methyl and ethyl, respectively) whereas in DBD4 and DBD5 we replaced a hydrogen by electron-accepting groups (fluorine and trifluoromethyl, respectively). Regardless of the type of replacement considered, our general result is that most of the barriers are in the range of 1–5 kcal/mol, regardless of the specific derivative considered. As more specific features, we can observe that the barriers corresponding to the HB formed by the residue ‘H2’ of the DBD derivatives are overall larger than those related to the hydrogen-bonds formed by ‘H4’, which suggests that ‘H2’ is the most stable binding site between DBD species and the model cell membranes considered in this work. Among the five DBD species analyzed we can observe that, regarding the ‘H2’ site of DBD, interactions of its derivatives with DOPC are about 10% stronger that those with DOPS but when ‘H4’ is concerned, the strength of its HB with DOPC is weaker than those with DOPS only when the tail groups ‘O22-32’ are considered. Nevertheless, the barriers of ‘H4’ to head groups are of similar size for both DOPC and DOPS. Further, we should remark a gross feature based on the class of substitution: derivatives DBD2 and DBD3 (where the -H group of the original DBD1 was replaced by electron-donating groups) show similar free-energy barriers and close to the values obtained for DBD1, whereas derivatives DBD4 and DBD5 (where the -H group of the original DBD1 was replaced by electron-accepting groups) also show similar free-energy barriers but less similar to the values obtained for DBD1. Finally, the binding of DBD with cholesterol is revealed to be sensibly weaker than that to DOPC and DOPS.

**Figure 7:**
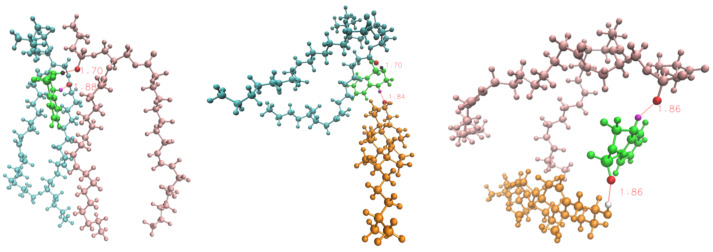
Snapshots of relevant configurations between benzothiadiazine derivative DBD4 (green) and their partner hydrogen-bonding sites. Lipid molecules: DOPS (pink), DOPC (cyan), cholesterol (orange). Specific sites: DBD4-H2 (black), DBD4-H4 (magenta), oxygen atoms in DBD4, DOPS, DOPC and cholesterol are depicted in red whereas hydrogen atom in cholesterol is depicted in white. Typical hydrogen-bond distances are indicated in red. This figure has been created by means of the “Visual Molecular Dynamics” package [81].

With the aim of a better understanding of the geometrical shape of the HB established between DBD and lipid species and as a sort of example, we report in Figure 7 a series of three snapshots describing the simultaneous binding of DBD4 with a few counterparts: so, we can observe that DBD4’s ‘H2’ and ‘H4’ are able to bridge oxygens ‘O13’ and ‘O14’ of DOPC and ‘O22-32’ of DOPS (A), also ‘O22-32’ of DOPC and ‘O’ of the hydroxyl group of cholesterol and finally ‘O13’ and ‘O14’ of DOPS and ‘O’ of the hydroxyl group of cholesterol. This remarkable bridging properties of DBD4 are qualitatively similar to those of DBD1. Both species, and to some extent all of DBD derivatives, can also form closed-ring structures (see Ref. [28], Figure 6). The bridging bonds highlighted here are quite similar to the HB structures observed in tryptophan [80] and melatonin absorbed at cell membrane surfaces [53].

#### 3.2.3. Time-Dependent Atomic Site–Site Distances

**Figure 8:**
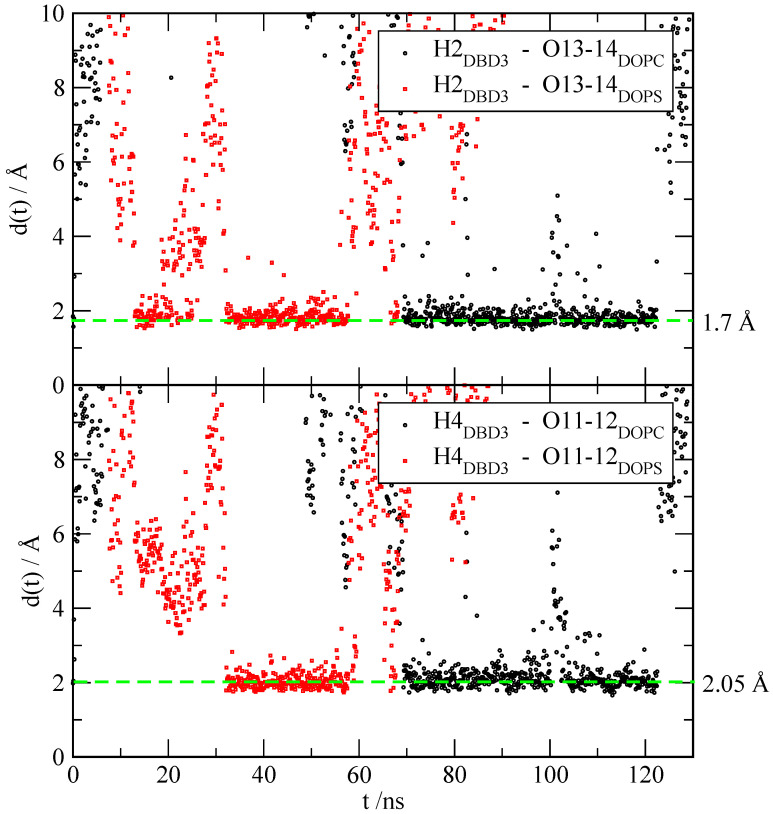
Time evolution of distances between selected sites of DBD3 (‘H2’, ‘H4’) and their partner oxygen sites of DOPC and DOPS.

Once the local structures of the DBD derivatives have been fully evaluated, we make an estimation of the HB dynamics by computing the average lifetime of some of the HB reported by RDF. Other typical MD properties involving time-correlation functions such as power spectra [82,83], relaxation times or self-diffusion coefficients [84,85] that were considered in previous studies, are out of the scope of this paper and have not been considered here. We display the time evolution of selected atom–atom distances d(t) in Figure 8 only for the pairings of ‘H2’ of DBD3 and sites ‘O13’ and ‘O14’ of DOPC and DOPS (top panel) and for ‘H4’ of DBD3 and sites ‘O11’ and ‘O12’ of DOPC and DOPS (bottom panel), as a sort of example. The full set of averaged values are reported in Table 3. We have selected in Figure 8 representative intervals (of more than 100 ns) from the full MD trajectory of 600 ns where the pattern of formation and breaking of HB is clearly seen, including a large extent of fluctuations. This means that such patterns have been systematically observed throughout the whole trajectory.

We can observe that typical HB distances of 1.7 and 2.05 Å are reached. Sites ‘O13’ and ‘O14’ (and ‘O11’ and ‘O12’) of DOPC and DOPS have been averaged given their equivalence. Typical HB lifetimes can vary enormously, between short lived HB of less than 1 ns (DBD1 with cholesterol) up to long-life HB of more than 70 ns (DBD2 with the head-group of DOPC, i.e., sites ‘O13-14’). As general trends, we can highlight that (1) sites ‘H2’ of the benzothiadiazine derivatives are able to form much longer lived HB than sites ‘H4’, especially for the DOPS species and (2) DBD–cholesterol hydrogen bonds have rather short lifetimes in the range of 1–10 ns. A closer look indicates that the longest living HB established between DBD and cholesterol are those composed by cholesterol’s hydrogen as donor and oxygens of DBD as acceptors, about twice longer that HB formed by hydroxyl’s oxygen of cholesterol and hydrogen ‘H4’ of DBD derivatives. For the sake of comparison, we should remark that the typical lifetime of hydrogen-bonds in pure water has been estimated to be of the order of 1 ps [86]. Finally, we should indicate that the shorter lifetimes reported in a previous work where DBD1 was studied [28] must be attributed to the shorter trajectories considered there and, especially, to the fact that some lifetimes were estimated without taking into account short-lived breaking and reformation of HB, as we did in the present work.

## 4. Conclusions

We report results from molecular dynamics simulations of benzothiadiazine derivatives embedded in a phospholipid bilayer membrane formed by 200 lipid molecules with concentrations of 56% of DOPC, 14% of DOPS and 30% of cholesterol in aqueous potassium chloride solution using the CHARMM36m force field. Starting from a standard 3,4-dihydro-1,2,4-benzothiadiazine-1,1-dioxide molecule we have designed in silico four derivatives based on the replacement of a single hydrogen atom by two different classes of chemical groups, one of them electron-donating groups (methyl and ethyl) and another one by electron-accepting groups (fluorine and trifluoromethyl). The electronegativity of these two groups is very different: whereas the electronegativity of electron-donating groups is smaller than that of hydrogen atoms, the electronegativity of electron-accepting groups is larger. In this paper, the electronegativity of methyl and ethyl groups, being smaller than that of hydrogen has the inductive effect of electron donation, which will increase the electron density of the DBD molecule to a certain extent. On the contrary, fluorine and trifluoromethyl will reduce the electron density of the molecule. When the hydrogen of the C-H bond in the DBD molecule is replaced by a substituent, the electron density distribution of the molecule changes, which has significant impact on the formation of hydrogen bonds between the drug and cell membrane components, as it has been reported in the present work.

As a gross feature, the same class of chemical groups produce similar effects on the HB between DBD and cell membranes, whereas different types tend to produce overall opposite effects. With this kind of study our aim is to elucidate the effects of different chemical groups on DBD–cell interactions. Our analysis is based on the computation of the local structures of the DBD derivatives when associated to lipids, water and cholesterol molecules. After the systematic analysis of meaningful data, we have found that the location of DBD at the interface of the membrane is permanent. We have computed RDF defined for the most reactive particles, especially hydrogens ‘H2’ and ‘H4’ and oxygens ‘O11-12’ of DBD (see Figure 1) correlated with sites of lipids and cholesterol able to form HB with DBD. All RDF have shown a strong first coordination shell and a weak second coordination shell for all DBD–lipid structures. The first shell is the signature of HB of lengths between 1.7 and 2.1 Å, in overall good agreement with experimental measurements [65] for comparable small-molecules at interfacial membranes.

The analysis of PMF of DBD–lipid interactions has revealed free-energy barriers of the order of 1–3 kcal/mol (Table 2), with the largest barriers corresponding to hydrogen bonds between DBD’s ‘H2’ site and oxygens sites of DOPC and DOPS; however, it has been observed that DBD derivatives are able to bind to cholesterol as well as the two classes of phospholipids, providing bridging connections that are able to locally estabilize and compactify the cell membrane, although the area per lipid and thickness of the whole membrane are not affected by the presence of the DBD species in any case. The influence of cholesterol has been especially noted in the weakening of DBD–lipid HB connections, which should be taken in consideration for the interaction of drugs with cell membranes from a pharmaceutical point of view. After a thorough analysis monitoring relative distances between tagged sites of DBD and lipids we have estimated the lifetime of HB by averaging data from the 600 ns MD trajectories to range in between 1 and 70 ns.

## Figures and Tables

**Table 1:** Area/lipid *A* and thickness Δz of the systems simulated in this work, given in Å${}^{2}$ and Å units, respectively. Estimated errors based on standard deviations correspond to the last significant figures, i.e., ±0.01 in each case.

DBD Derivative	*A*	Δz
DBD1	52.18	43.04
DBD2	52.20	43.02
DBD3	52.23	42.98
DBD4	52.19	43.02
DBD5	52.23	42.97

**Table 2:** Free-energy barriers ΔW (in $k_{B}T$) from reversible work calculations for the binding of DBD to cholesterol, lipids and water. In order to quantify the height of all barriers, 1 $k_{B}T=0.596$ kcal/mol. Labels as indicated in Figure 1. Estimated errors of ±0.1 in all cases.

DBD Site	Lipid Site	ΔW				
		DBD1	DBD2	DBD3	DBD4	DBD5
H2	O13-14 DOPC	4.2	4.0	4.0	4.2	5.2
H2	O22-32 DOPC	3.4	3.2	2.5	4.4	3.7
H2	O13-14 DOPS	4.2	3.8	5.0	4.7	4.7
H2	O22-32 DOPS	2.8	3.5	3.3	3.0	2.2
H4	O11 DOPC	2.0	2.5	2.4	1.9	3.1
H4	O22-32 DOPC	1.6	2.2	1.9	2.3	2.3
H4	O11 DOPS	2.3	1.2	3.5	1.8	3.8
H4	O22-32 DOPS	1.8	1.6	2.0	4.0	3.9
H4	O Cholesterol	1.2	3.2	2.9	2.4	3.4
O11-12	H Cholesterol	1.8	2.4	2.2	2.6	2.3

**Table 3:** Averaged distances (in Å) between selected sites of DBD and the membrane. Continuous time intervals (τ, in ns) have been obtained from averaged computations along the 600 ns trajectory. Labels as indicated in Figure 1. Estimated errors of ±0.1 in all cases.

DBD Site	Lipid Site	Distance	$\tau_{\mathrm{DBD}1}$	$\tau_{\mathrm{DBD}2}$	$\tau_{\mathrm{DBD}3}$	$\tau_{\mathrm{DBD}4}$	$\tau_{\mathrm{DBD}5}$
H2	O13-14 DOPC	1.7	67.4	73.7	63.2	28.4	38.5
H2	O22-32 DOPC	1.8	20.4	11.8	12.3	19.4	24.6
H2	O13-14 DOPS	1.7	6.9	8.8	27.0	9.6	9.7
H2	O22-32 DOPS	1.8	1.4	1.9	2.3	3.1	0.9
H4	O11-12 DOPC	1.9	42.5	64.4	61.5	15.6	35.9
H4	O22-32 DOPC	2.0	16.5	14.0	13.0	16.6	28.4
H4	O11-12 DOPS	1.9	1.3	1.9	24.8	1.9	6.4
H4	O22-32 DOPS	2.0	1.8	1.4	0.9	2.6	1.9
H4	O Cholesterol	2.0	0.5	3.4	4.4	3.7	3.6
O11-12	H Cholesterol	1.9	4.6	4.5	6.6	10.3	6.0

## Data Availability

Not applicable.
